# Diplomas for Sale

**Published:** 1870-02

**Authors:** 


***Diplomas for Sale.***

SOME time last year, we referred to the fact that an agency for a certain "University" in Philadelphia, had been established in this city for the sale of diplomas of M.D., and we had authority for saying that several persons in this city and the surrounding country had availed themselves of the opportunity for their purchase. We have in our possession original documentary evidence of this fact.

It becomes now our unplesant duty, as a patriotic citizen of Chicago, to say that our enterprising neighboring city of Milwaukee has, for once, beaten Chicago at this game.

From a rejoicing applicant, we have received the following papers which fully explain themselves. (We omit the name of the philanthropic "agent," as we do not propose to advertise him except at our regular rates, as established by the publisher).

The first is a card, with this on the obverse:

THE MILWAUKEE MEDICAL AND SURGICAL INSTITUTE, (Chartered by the Legislature of Wisconsin), office, corner of East Water and Mason Streets.

*Collegiate Agency.* — This agency has been established for the purpose of giving such information as is necessary, before taking any of the learned degrees in arts, science, law and medicine, and represents some of the best universities in America and Europe.

By or through its recommedation, the degrees of A.M. (Master of Arts), A.B. (Bachelor of Arts), LL.D. (Doctor of Laws), M.D. (Doctor of Medicine), D.D.S. (Doctor of Dental Surgery), etc , may be legitimately conferred.

For particulars, address the Principal.

MILWAUKEE, WIS.

And this on the reverse:

"Many States have passed laws prohibiting physicians, who are not graduates, from practicing medicine, collecting fees, or giving evidence in court on medical matters. All the other States will soon pass similar laws. Similar qualifications are required of surgeons of the U. S. Army. In view of the agitation of this question, and the facts that no physician without a diploma can be fully recognized as a *bona fide* member of the profession, and that it is a passport to good fellowship with his professional brethren and the confidence of the community, every practicing physician should be able to produce this badge of his profession."

The next is a printed circular, with same heading:

[CONFIDENTIAL.]

DEAR SIR: — In reply to frequent inquiries on the subject, by physicians, the following is published for the information and guidance of those seeking diplomas through the agency of this institution:

1. It is not the intention or object of the agency to confer degrees upon improper or incompetent persons.

2. The principal object of the agency is to secure, for the schools in which we are interested, students prepared to attend their second course of lectures. All schools feel a pride in exhibiting a large graduating class. Those schools which we represent being among the best in the country, the advantage is mutual.

3. The requirements for graduation, in all these schools, are three years' study, and two full courses of lectures.

4. In cases where it seems justifiable, some of the schools have decided to depart from this last rule, in so far as to confer diplomas upon physicians who, although they may not have attended two full courses of lectures, have had sufficient experience in the study and practice of medicine to compensate for the deficiency. Five years' reputable practice without having attended lectures, or one course of lectures and three years' reputable practice, are considered as an equivalent. No one can doubt but that a physician who has been engaged in active practice for five or more years, is as much entitled to the degree of M.D., as three-fourths of the raw students who are graduated by all universities at the end of their second course.

This action of the schools, however, being "irregular," and rival schools always ready to seize upon any pretext to injure their reputation, it is necessary that the faculties should not be approached upon the subject; but all business of the kind must be strictly confidential, and transacted through their private agents, of whom we are the only one in the North-west.

5. It will be seen that we do not confer the degree, but act merely as a go-between the candidate and the schools. The transaction is closed when the diploma is conferred; and those who apply for and receive this information are bound in honor to regard it as confidential, whether they avail themselves of the privilege or not, and their application, on our part, will ever be held in sacred confidence.

6. EXAMINATIONS. — Examination of candidates for graduation may be by actual oral examination, by us, at the institute, in which such questions, etc., are propounded upon therapeutics, surgery, obstetrics, materia medica, chemistry, anatomy, etc., as would be asked by the faculty in the "green room."

Otherwise the examination may be merely *pro forma*; the presentation to us of proper evidence that the applicant has attended one course of lectures and practiced three years; or has practiced five years; a certificate from any medical society, or any two well known or respectable physicians (graduates) that he is a competent person to engage in the practice of medicine, will be accepted by us as *prima facie* evidence that he is properly qualified and we will file these credentials with the faculty, report that "we have examined the applicant, etc., and recommend him for graduation."

The third is an autograph letter, with same heading:

DEAR SIR: — Yours received. I can procure you a degree of M.D., without your leaving home, from one of the medical universities of Philadelphia. You must leave the selection of the school to me, as all of them do not grant degrees in this way. It will be from a respectable, well established and chartered school, signed and sealed by the faculty and officers, and your name enrolled on the college books — every thing the same exactly as if you went there and attended a course of lectures, and graduated; and in case of any difficulty, you may refer to the faculty, or in your cards.

The entire expense, including every thing, will be $150.00; of which amount, $50 must accompany the application, and when the diploma is sent, which will be within two weeks, it will be C. O. D., the balance $100; or you can send the full amount at once, and it will be sent.

The certificates, etc., may be dispensed with, on my recommendation but it will facilitate matters if you can send them. Yours truly,

——.

We beg leave to add only, at present, that medical legislation, thus far does not appear to have elevated the profession to any very admirable extent.

